# Draft Genome Sequences of Eight Campylobacter volucris Isolates from Freshwater Sources in Victoria, Australia

**DOI:** 10.1128/MRA.01086-20

**Published:** 2021-01-07

**Authors:** Agnus M. Davis, David. T. McCarthy, Dieter M. Bulach, Rebekah Henry

**Affiliations:** a Environmental and Public Health Microbiology Laboratory (EPHM Lab), Department of Civil Engineering, Monash University, Clayton, Victoria, Australia; b Microbiological Diagnostic Unit Public Health Laboratory, The Peter Doherty Institute, The University of Melbourne, Parkville, Victoria, Australia; University of Delaware

## Abstract

*Campylobacter* spp. can survive and be transmitted from a range of environments. Here, we examine eight draft genome sequences of Campylobacter volucris, identified as part of an examination of waterborne *Campylobacter* species. This is the first report of environmental survival of *C. volucris* outside gull species.

## ANNOUNCEMENT

*Campylobacter*-associated infection is often spread to humans via consumption of poultry, beef, lamb, or pork products (1). However, there are increasing reports of environmental pathways (water, soil) to disease transmission (2–5). *Campylobacter* species can be introduced into these environments through direct defecation by zoonotic disease vectors (e.g., birds, dogs, livestock). For example, sampling of the cloaca of black-headed gulls (Larus ridibundus) in Sweden revealed carriage of a Campylobacter lari*-*like strain (6). Further DNA analysis identified the bacterium to be a distinct *Campylobacter* species, Campylobacter volucris. To date, *C. volucris* has been minimally reported within the literature, with eight published genome sequences primarily associated with black-headed gulls (7).

In this study, waterborne strains were isolated from five freshwater locations across Greater Melbourne (Table 1). The samples underwent membrane filtration onto 0.45-μm cellulose nitrate filters (Sartorius, Germany). Isolation was undertaken as described in Australian/New Zealand standard 4276.19:2001 (AS/NZS, 2001), with membranes placed into 25 ml Preston broth and resuscitated aerobically for 2 h at 37°C, prior to the addition of *Campylobacter* selective supplement (Oxoid, UK) and microaerobic incubation (5% O_2_, 10% CO_2_, and 85% N_2_) for 48 h at 42°C. Postenrichment (48 h at 42°C), 2 μl of each sample was plated onto modified charcoal-cefoperazone deoxycholate agar (CCDA)-Preston medium and incubated for 48 h at 42°C (Oxoid). The colonies underwent biochemical confirmation using the Oxoid Biochemical Identification System (OBIS) Campy kit, followed by selection on horse blood agar (HBA) (Oxoid) and microaerophilic growth for 48 h at 42°C.

**TABLE 1:** Australian Campylobacter volucris isolation, sequencing, and genome assembly data

Parameter	Data for strain:							
	MON0058	MON0078	MON0115	MON0166	MON0180	MON1184	MON1267	MON2072
Sequencing technology	Illumina NextSeq 500	Illumina NextSeq 500	Illumina NextSeq 500	Illumina NextSeq 500	Illumina NextSeq 500	Illumina NextSeq 500	Illumina NextSeq 500	MGITech DNBSEQ-G400
Library chemistry	Nextera XT	Nextera XT	Nextera XT	Nextera XT	Nextera XT	Nextera XT	Nextera XT	MGIEasy DNA FS
Sequencing chemistry	NextSeq 500 v2	NextSeq 500 v2	NextSeq 500 v2	NextSeq 500 v2	NextSeq 500 v2	NextSeq 500 v2	NextSeq 500 v2	DNBSEQ high-throughput sequencing set FCL
Read length (bp)	PE150	PE75	PE150	PE75	PE75	PE150	PE150	PE100
No. of reads	2,404,222	3,743,990	913,212	3,969,814	3,316,282	1,203,542	1,195,848	5,627,484
No. of contigs	36	292	61	281	340	28	64	27
Genome length (bp)	1,486,257	1,467,362	1,508,747	1,548,798	1,413,139	1,497,020	1,637,888	1,519,422
*N*_50_ (bp)	83,244	9,015	66,645	11,468	6,494	109,393	73,782	128,448
Coverage (×)	220	174	83	184	147	112	113	364
GC content (%)	30.3	33.4	30.8	33.6	32.7	30.7	30.3	28.8
SRA accession no.	SRX9038520	SRX9038521	SRX9038522	SRX9038523	SRX9038524	SRX9038525	SRX9038526	SRX9038527
BioSample accession no.	SAMN15944590	SAMN15944591	SAMN15944592	SAMN15944593	SAMN15944594	SAMN15944595	SAMN15944596	SAMN15944597
BioProject accession no.	PRJNA660282	PRJNA660282	PRJNA660282	PRJNA660282	PRJNA660282	PRJNA660282	PRJNA660282	PRJNA660282
Source	Rural river	Rural river	Rural river	Rural river	Rural river	Rural river	Rural river	Urban river
GPS location	37°42′25.1″S, 145°19′19.5″E	37°42′25.1″S, 145°19′19.5″E	37°42′25.1″S, 145°19′19.5″E	37°40′25.3″S, 145°29′20.1″E	37°42′25.1″S, 145°19′19.5″E	37°45′17.7″S, 145°40′46.8″E	37°45′17.7″S, 145°40′46.8″E	37°47′38.2″S, 145°00′06.8″E

Genomic DNA was extracted using the RBC genomic DNA extraction kit (Real Biotech, Taiwan). The DNA quality and quantity were assessed using a Qubit fluorometer (Thermo Fisher Scientific, Waltham, MA, USA) following the manufacturer’s instructions. Sequencing libraries were prepared using 1 ng DNA for the Nextera XT kit (Illumina, USA) and 100 ng DNA for the MGIEasy DNA FS kit (MGI, China). Sequencing of the Nextera XT libraries was conducted on the NextSeq 500 platform (Illumina) using v2 2 × 150-bp paired-end (PE) chemistry, while the MGI libraries underwent sequencing on an MGITech DNBSEQ-G400 (MGI) instrument using the 150PE DNBSEQ high-throughput sequencing set FCL v2 chemistry (MGI). Sequence data were quality processed, analyzed, and *de novo* assembled using the tools within the Nullarbor v2.0 pipeline (8). Default parameters were applied to all software contained therein unless otherwise specified.

The draft genome sequence and assembly statistics are provided in Table 1. *De novo* assembly achieved an overall coverage of 135× (±43×), with the strains having an average GC content of 31% (±2%) and size of 1.5 Mb (±62 kb). These characteristics are similar to those previously reported for *C. volucris* isolates.

This study reports the draft genome sequence for eight *C. volucris* isolates from waterways in Victoria, Australia; this is the first non-host-associated isolation of this species. Thus, this report extends our understanding of the genetic diversity within the species and is the first demonstration that this organism has the capacity to exist in environmental waters. We also note that black-headed gulls are not part of the indigenous fauna of the region, indicating a broader avian host range, possibility associated with other waterfowl species.

### Data availability.

The genome sequences and raw data have been deposited in GenBank within BioProject accession number PRJNA660282, under the accession numbers outlined in Table 1.
